# Supplementary material for: Electrospinning and Post-Spun Chain Conformations of Synthetic, Hydrophobic Poly(α-amino acid)s
Source: Polymers (Basel). 2020 Feb 4;12(2):327. doi: 10.3390/polym12020327 (PMC7077440; doi:10.3390/polym12020327)
Supplement: Supplementary file 1 [file polymers-12-00327-s001.pdf]

Supplementary Information for

Electrospinning and Post-spun Chain Conformations of Synthetic, Hydrophobic Poly(α-amino acid)s

Kesavan Devarayan[a], Souta Nakagami[b], Shuichi Suzuki[c], Ichiro Yuki[c,d], Kousaku Ohkawa[b,c,e]*

[a] Department of Basic Sciences, College of Fisheries Engineering, Tamil Nadu Dr. J. Jayalalithaa Fisheries University, Vettar River View Campus, Nagapattinam 611 002, India.

[b] Division of Synthetic Polymers, Institute of High Polymer Research, Faculty of Textile Science and Technology, Shinshu University, Tokida 3-15-1, Ueda 386-8567, Japan.

[c] Department of Neurological Surgery, University of California, Irvine, 200S Manchester St., Suite 210, Orange, CA92868, USA.

[d] Department of Neurosurgery, The Jikei Univeristy Hospital, 3 Chome-25-8 Nishishinbashi, Minato City, Tokyo 105-8461, Japan

[e] Division of Biological and Medical Fibers, Institute for Fiber Engineering, Interdisciplinary Cluster for Cutting Edge Research, Shinshu University, Tokida 3-15-1, Ueda 386-8567, Japan.

* Corresponding author: Tel. +81 268 215573; Fax. +81 268 5571.

Email: kohkawa@shinshu-u.ac.jp

S1. Molecular weight determination by *N*-terminus labeling method

Synthesis of dinitrophenyl (DNP)-L-amino acids

L-Ala (0.89 g, 10.0 mmol), L-Val (1.17 g, 10.0 mmol), and L-Leu (1.31 g, 10.0 mmol) were taken separately and dissolved in distilled water (36 mL) containing Na_2CO_3 (2.00 g, 18.9 mmol). The reaction mixture was warmed to 40 °C, then added DNFB (1.83 g, 10.0 mmol) and 4 mL of acetone and stirred under darkness for 90 min. The reaction mixture was washed with diethyl ether, and then acidified using 4 M HCl. The product was extracted using ethyl acetate and dried over anhydrous Na_2SO_4. The solvent was condensed after filtering off Na_2SO_4. The product was obtained by scratching with hexane and the crude product was re-crystallized from ethyl acetate/hexane. DNP-L-Ala (**9**): Yellow crystals; Yield: 1.90 g, 74.5 %. DNP-L-Val (**10**): Yellow crystals; Yield: 2.08 g, 73.4 %. DNP-L-Leu (**11**): Yellow oily substance; Yield: 2.81 g, 94.5%.

DNP-L-Leu (**11**) was dissolved in diethyl ether then treated with cyclohexyl amine (0.94 g, 9.48 mmol). The product, DNP-L-Leu • cyclohexyl amine (DNP-L-Leu • CHA) (**13**) was filtered off and recrystallized from methanol/diethyl ether. Yellow crystals; Purified yield: 2.15 g, 54.2%.

*Determination of molecular extinction coefficient for DNP-L-Ala (**10**), DNP-L-Val (**11**), and DNP-L-Leu • CHA (**13**)*

DNP-L-Ala (**9**), DNP-L-Val (**10**), and DNP-L-Leu • CHA (**12**) were independently

dissolved in phosphate buffer (pH 6.7) to prepare solutions having the following concentrations 0.10 mM, 0.07 mM, 0.04 mM, and 0.01 mM, respectively. The absorbance for DNP was observed at 360 nm using a Hitachi U-2000A spectrophotometer. The molecular extinction coefficients were calculated according to Beer-Lambert's law and the values were 17,100, 15,100, and 15,600 for DNP-L-Ala (**9**), DNP-L-Val (**10**), and DNP-L-Leu • CHA (**12**), respectively.

N-terminus labeling reaction

5.0 mg of poly(L-Ala) (**2**) was dispersed in 5 mL of benzene, and each 5.0 mg of poly(L-Val) (**4**) and poly(L-Leu) (**6**) were swollen in 5 mL of benzene. The three samples were sonicated for 60 min at room temperature. Poly(L-Val) and poly(L-Leu) were finally completely dissolved, on the other hand, poly(L-Ala) was not completely dissolved in which fine particles remained. Since the *Dp*s of three poly(amino acid)s were unknown, the stoichiometry of the labeling reagent DNFB was fixed at 1.0 eq. mmol towards the *N*-terminal residues, assuming that the average *Dp* values are *Dp* = 50 and 100 ("Hypothetical *Dp*"). DNFB was added to the poly(amino acid) solutions (or fine dispersion) with 10 eq.mol of TEA, and the labeling reaction was continued under darkness for 6 hr with vigorous shaking. The labeled poly(amino acid)s were recovered by evaporation and immediately re-dissolved (or re-dispersed) in reaction solvents followed by re-precipitation using diethyl ether. The labeled poly(amino acid)s were then thoroughly washed with diethyl ether for complete removal of unreacted DNFB.

Amino acid analysis

Pre-determined weights of the labeled poly(amino acid)s (ca. 1.0–1.5 mg) were placed in small ampoule bottles and once dissolved in TFA (200 μL), then diluted with equal volume of concentrated hydrochloric acid (amino acid analysis grade). The bottles were sealed under reduced pressure, and the samples were incubated at 110 ^{o}C for 24 h. All mixtures were yellowish transparent through the complete hydrolysis reaction. The bottles were opened and the solvents were removed under *vacuo*. Then the samples were dissolved in citrate buffer (pH 2.2, 250 μL) (Wako Pure Chemical, Ind., Osaka, Japan), and subjected to automated amino acid composition analysis using a Shimadzu LCVp10 analyzing system. The eluted amino acids were detected by a post-column labeling with *o*-phthalaldehyde.

Quantification of DNP-amino acid as N-terminal residue

The hydrolyzed samples (100 μL) were diluted with phosphate buffer by 20-fold at the pre-determined concentrations appropriate for the absorbance measurements using a quartz cell with path length of 1.0 cm. Prior to the measurements of the samples, molecular extinction coefficients (at 360 nm) of N^{α}-DNP-L-Ala, -L-Val, and -L-Leu were determined using authentic samples, which have been separately synthesized from DNFB and free L-amino acids. The amount of N^{α}-DNP-amino acid in the hydrolyzed samples was quantified on the basis of observed adsorption at 360 nm.

Determination of M_n and Dp by N-Terminus Labeling

The average Dp^N and M_n^N were calculated by the following equations (Eq. S1 and S2) and the values were given in table S1.

$$Dp^N = \frac{F}{F'} \times 10^5 \qquad \text{(S1)}$$

$$M_n^N = Dp^N \times W_r \qquad \text{(S2)}$$

where, *F* and *F'* were total amino acid found (in μmol) and DNP-amino acid found (in nmol), respectively. W_r was the residue weight of amino acid.

Two hypothetical *Dp*s, *Dp* = 50 and 100 were considered for *N*-terminus labeling, where DNFB was obviously excess towards hypothetical amounts of *N*-terminus. The estimated Dp^N values were mostly close to each other for both hypothetical *Dp*s, indicating saturation of *N*-termini by DNP-labeling.

This method is convenient for routine estimation of *Dp* and M_n of insoluble poly(amino acid)s, however, the methodology involves an observed experimental error of approximately 10–15%. The degree of experimental error is dependent on both of the labeling yield and the amino acid composition analysis. Currently there is no procedure available to confirm the quantitative labeling of *N*-termini. Therefore, the two hypothetical *Dp*s for addition of DNFB in excess molar amount towards the *N*-termini labeling are effective, if the observed values from two hypothetical *Dp*s exhibited closer values within the experimental error.

S2. Molecular Weight Determination of Poly(Gly) by Limiting Viscosity Comparison

Poly(L-Val) and poly(Gly) were dissolved in TFA and the viscosity measurements were performed using a Ubbelohde viscometer at 25 ^{o}C.

*Estimation of Dp of Poly(Gly) (**8**) on Comparison with Poly(L-Val) (**4**)*

The average molecular weight (M_n^V) and degree of polymerization (Dp^V) for poly(Gly) (8) was determined by the following equations (Eq. S3 and S4) and the values were given in table S2.

$$Dp^V = Dp^N \times \frac{V^{Gly}}{V^{L\text{-}Val}} \quad (4)$$

$$M_n^V = Dp^V \times W_{Gly} \quad (5)$$

where, Dp^V was the degree of polymerization estimated from ratio between the limiting viscosities of poly(Gly) (V_L^{Gly}) and poly(L-Val) ($V_L^{L\text{-}Val}$).

One of the obstacles that limit the studies on hydrophobic poly(amino acid)s is the molecular weight determination. Dissolution of the poly(amino acid)s is a prerequisite for both viscometry and GPC, whilst among the various kinds of amino acid residues, depending on the average degree of polymerization (Dp), poly(amino acid)s are often insoluble in organic acids for viscometry and in aprotic solvents for GPC.

In the present study, N-terminus labeling method was employed for estimation of Dp and number average molecular weights (M_n) of three kinds of hydrophobic poly(amino

acid)s, namely, poly(L-Ala), poly(L-Val), and poly(L-Leu). The labeling reagent, 2,4-dinitrofluorobenzene (DNFB) has been developed by Sanger in 1940s [20], and since then this reagent has been widely used in biochemistry and polymer chemistry of proteins [21]. The complete hydrolysis of DNP-poly(amino acid)s after the labeling reaction liberates the N^{α}-DNP-aa as *N*-terminal residues, together with free amino acids as non-terminal (endo-) residues. Spectrophotometrical quantification of N^{α}-DNP-amino acid and conventional chemical quantification of the free amino acids allows to calculate the *Dp* and M_n of the subjected poly(amino acid)s, without separating N^{α}-DNP-amino acid from free amino acids.

Figures

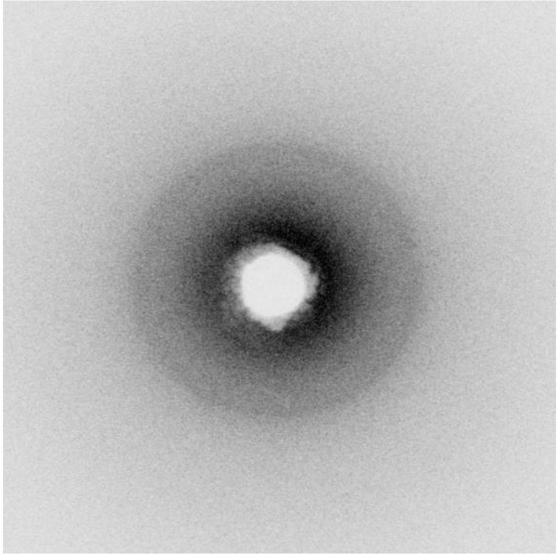

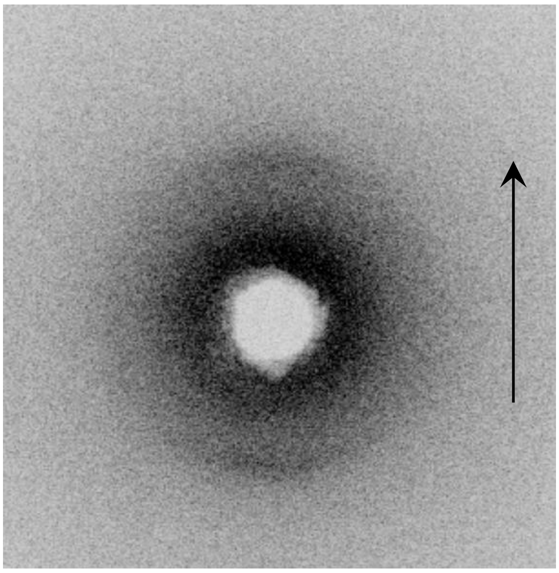

(b)

Fig. S1. WAXD pattern for pre-drawn (a) and drawn (b) films of poly(L-Ala). Drawn axis is indicated by an ‘upward arrow’.

Tables

Table S1. Determination of *Dp* and M_n by *N*-terminus labeling.

	$Dp^{a)}$	Sample weight (mg)	Amino acid found (μmol)	Abs at 360 nm	DNP-amino acid (nmol)	Dp^N	M_n^N
Poly(L-Ala) (**2**)	50	0.936	6.54	0.111	260	2520	179000
	100	1.01	8.96	0.137	343	2610	185000
Average		0.978	3.79	0.124	236	2570	184000
Poly(L-Val) (**4**)	50	0.935	4.26	0.022	1570	271	26900
	100	0.862	4.06	0.017	1300	312	30900
Average		0.899	4.16	0.020	1080	292	28900
Poly(L-Leu) (**6**)	50	1.01	2.38	0.056	146	1630	184000
	100	1.11	3.23	0.087	220	1470	166000
Average		1.06	2.81	0.067	183	1550	175000

[a)] Hypothetical *DP*.

Table S2. Determination of *Dp* and M_n of poly(Gly) by limiting viscosity comparison.

	Dp^N	$M_n{}^N$	$[\eta]_{TFA}$	Dp^V	$M_n{}^V$
Poly(L-Val) (**4**)	292	28900	0.352	-	-
Poly(Gly) (**8**)	-	-	0.186	502	15100
